# Cricopharyngeal peroral endoscopic myotomy for the treatment of a cricopharyngeal bar causing oropharyngeal dysphagia

**DOI:** 10.1055/a-2953-8922

**Published:** 2026-10-08

**Authors:** David Leonard, Darren Boone, Vinay Sehgal

**Affiliations:** 1 Gastroenterology 8964 University College London Hospitals NHS Foundation TrustLondonUnited Kingdom of Great Britain and Northern Ireland; 2 Faculty of Medical Sciences, Gastroenterological intervention 4919 University College LondonLondonEnglandUnited Kingdom of Great Britain and Northern Ireland

The cricopharyngeal muscle (CPM) is a key component of the upper oesophageal sphincter helping with swallowing and preventing aspiration. 1​ A cricopharyngeal bar (CPB) is a radiological description caused by CPM hypertrophy which limits adequate oesophageal distension. 2​ The causes may be idiopathic or secondary to neurodegenerative disorders, such as inclusion body myositis. Most patients are asymptomatic; however treatment may be required for those who exhibit symptoms including oropharyngeal dysphagia, regurgitation and deglutitive cough. Endoscopic treatments such as botulinum toxin injection and balloon dilation have been described but have varying clinical success. 3​ Cricopharyngeal peroral endoscopic myotomy (CP-POEM) is a new technique based on the principles of third-space endoscopy. 4​ ​5​

A 72-year-old woman was referred with a 2-year history of oropharyngeal dysphagia, following anterior cervical discectomy and fusion. Barium swallow ( Fig. 1 ) showed significant cricopharyngeal spasm, occluding 70% of the oesophageal lumen and demonstrated a CPB.

**Fig. 1:**
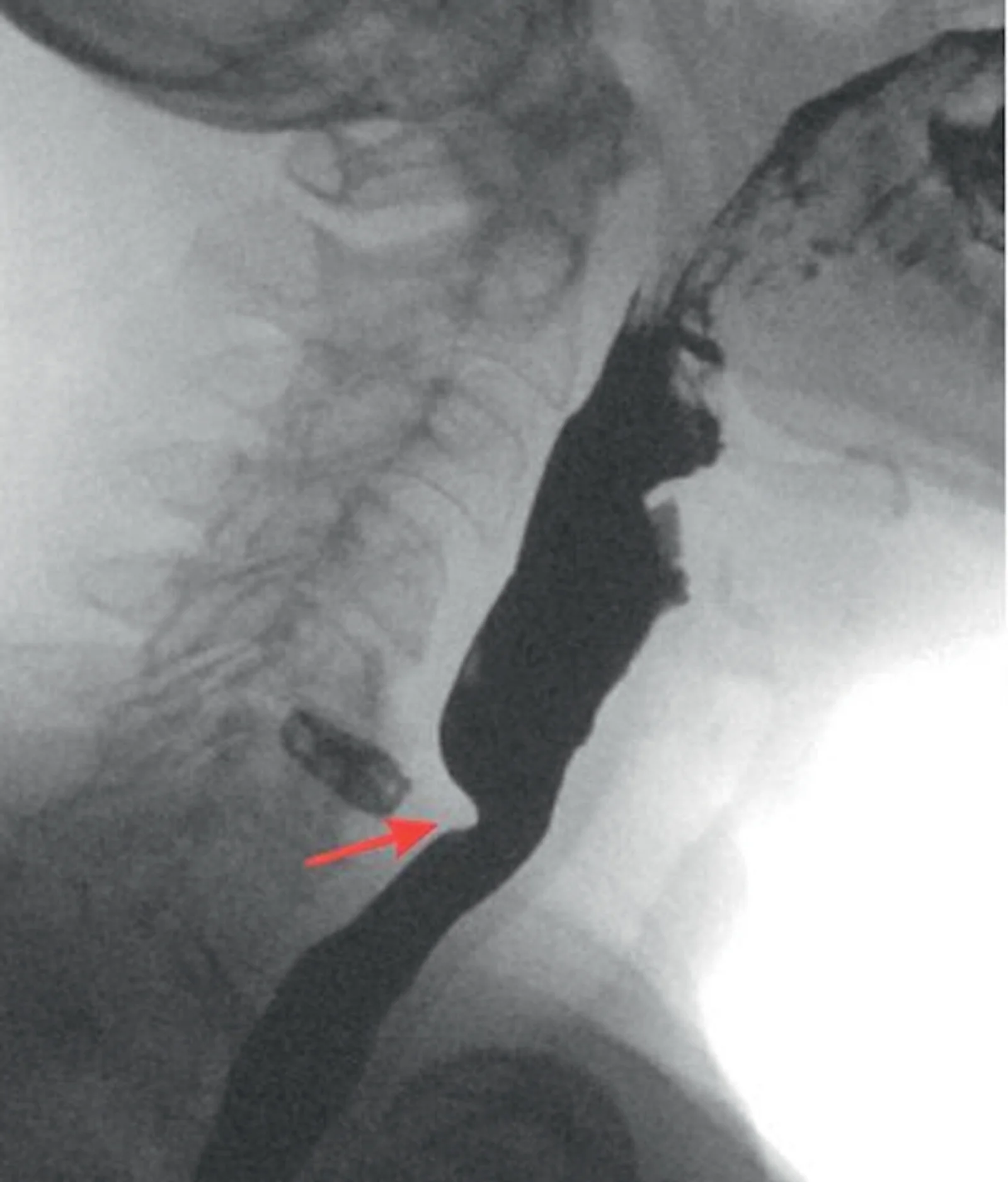
Pre-barium swallow showing the cricopharyngeal bar.

A CP-POEM was performed, as shown in Video 1 and Fig. 2 . The procedure initially involves the identification of the CPB. Next, a submucosal injection of indigo carmine and adrenaline (1:200,000), beginning in the piriform fossa, was performed, followed by mucosectomy (HYBRIDknife flex; ERBE, Germany) to allow entry of the endoscope (EG29-i10; PENTAX Medical, Tokyo, Japan) into the submucosal tunnel. The submucosal tunnel was extended 2 cm beyond the CPB. The CPB was confirmed within the tunnel based on a combination of measurements and tactile resistance upon the passage of the endoscope through the submucosal tunnel. A full-thickness myotomy 1 cm proximal and 2 cm distally to the CPM was then performed. Examination for any mucosal injuries was performed followed by spray injection of gentamicin (80 mg) into the tunnel. The tunnel entrance was closed using 8 mm Lockado clips (Micro-Tech Endoscopy). The patient had an uneventful recovery and had a complete improvement in dysphagia and resolution of CPB ( Fig. 3 ) on 3-month follow-up.

**Video 1** Demonstration of cricopharyngeal peroral endoscopic myotomy.


**Fig. 2:**
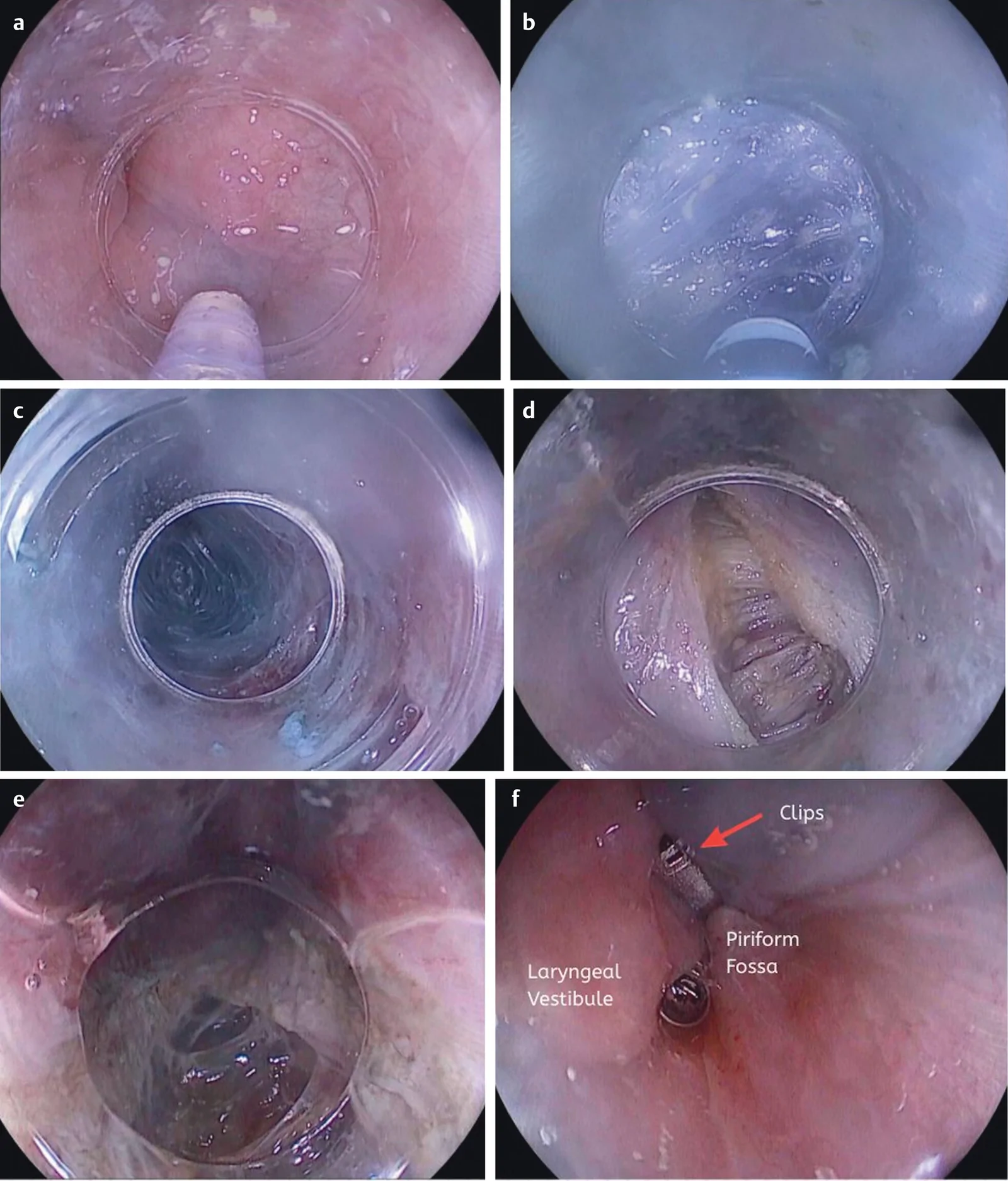
Images showing the sequential steps of CP-POEM. ( **a** ) Submucosal injection is made in the piriform fossa. ( **b** ) Submucosal tunnelling. ( **c** ) Completion of the submucosal tunnel. ( **d** ) Identification of the cricopharyngeal muscle. ( **e** ) Post myotomy. ( **f** ) Closure of tunnel entrance.

**Fig. 3:**
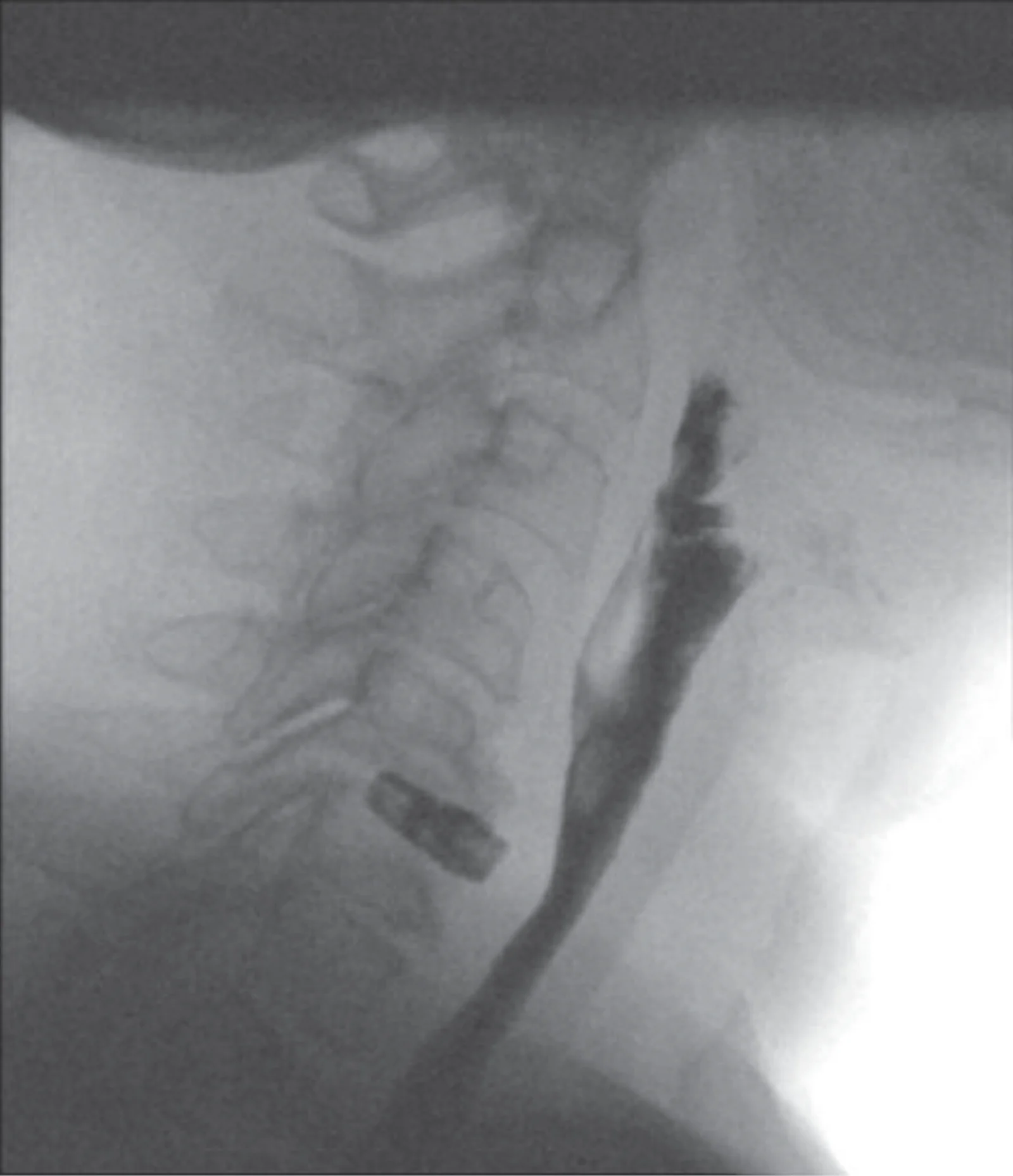
Post-treatment barium swallow showing good passage of barium and resolution of the previous bar.

Endoscopy_UCTN_Code_TTT_1AO_2AP

## Declaration of GenAI Use

The authors confirm that no generative artificial intelligence was used in the preparation of this manuscript.

## References

[1] Cook I J Cricopharyngeal function and dysfunction Dysphagia 1993 8 244 251 10.1007/bf01354546 8359046

[2] Cook I Cricopharyngeal bar and zenker diverticulum Gastroenterol Hepatol (N Y) 2011 7 540 PMC3264939 22298991

[3] Albeniz E Estremera-Arevalo F Cricopharyngeal achalasia and upper oesophageal endoscopic myotomy (CP-POEM) Best Pract Res Clin Gastroenterol 2024 71 101937 10.1016/j.bpg.2024.101937 39209419

[4] Elmunzer B J Moran R A Peroral endoscopic myotomy for cricopharyngeal bar VideoGIE 2020 5 378 379 10.1016/j.vgie.2020.04.020 PMC7426955 32821871

[5] Norton B Papaefthymiou A Telese A Peroral endoscopic myotomy for the management of symptomatic cricopharyngeal bar (C-POEM): a case series and video demonstration Ann Gastroenterol 2024 37 251 254 10.20524/aog.2024.0856 PMC10927621 38481774

